# Scoliosis treatment using spinal manipulation and the Pettibon Weighting System™: a summary of 3 atypical presentations

**DOI:** 10.1186/1746-1340-14-1

**Published:** 2006-01-12

**Authors:** Mark W Morningstar, Timothy Joy

**Affiliations:** 1 Director of Research, The Pettibon Institute; 3416-A 57^th ^St Ct NW Gig Harbor WA 98335, USA; 2 Evergreen Spine & Posture Correction Center; 6615 6th Ave Tacoma, WA 98406, USA

## Abstract

**Background:**

Given the relative lack of treatment options for mild to moderate scoliosis, when the Cobb angle measurements fall below the 25–30° range, conservative manual therapies for scoliosis treatment have been increasingly investigated in recent years. In this case series, we present 3 specific cases of scoliosis.

**Case presentation:**

Patient presentation, examination, intervention and outcomes are detailed for each case. The types of scoliosis presented here are left thoracic, idiopathic scoliosis after Harrington rod instrumentation, and a left thoracic scoliosis secondary to Scheuermann's Kyphosis.

Each case carries its own clinical significance, in relation to clinical presentation. The first patient presented for chiropractic treatment with a 35° thoracic dextroscoliosis 18 years following Harrington Rod instrumentation and fusion. The second patient presented with a 22° thoracic levoscoliosis and concomitant Scheuermann's Disease. Finally, the third case summarizes the treatment of a patient with a primary 37° idiopathic thoracic levoscoliosis. Each patient was treated with a novel active rehabilitation program for varying lengths of time, including spinal manipulation and a patented external head and body weighting system. Following a course of treatment, consisting of clinic and home care treatments, post-treatment radiographs and examinations were conducted. Improvement in symptoms and daily function was obtained in all 3 cases. Concerning Cobb angle measurements, there was an apparent reduction in Cobb angle of 13°, 8°, and 16° over a maximum of 12 weeks of treatment.

**Conclusion:**

Although mild to moderate reductions in Cobb angle measurements were achieved in these cases, these improvements may not be related to the symptomatic and functional improvements. The lack of a control also includes the possibility of a placebo effect. However, this study adds to the growing body of literature investigating methods by which mild to moderate cases of scoliosis can be treated conservatively. Further investigation is necessary to determine whether curve reduction and/or manipulation and/or placebo was responsible for the symptomatic and functional improvements noted in these cases.

## Background

Idiopathic scoliosis is estimated to affect about 2–3% of adolescent females age 10–16 years [1-3]. Scoliosis is a postural deformity characterized as a lateral curvature of the spine greater than 10°, measured by the Cobb method on standing upright spine radiographs [4]. While most cases of scoliosis are classified as idiopathic [2], a minority of scoliosis cases are traced to structural anomalies [3], such as wedged vertebrae or abnormal soft tissue development.

In addition to lateral curvature, scoliosis is also recognized in the sagittal plane. One of the potential causes of sagittal plane scoliosis is Scheuermann's Disease. Scheuermann's Disease is characterized by wedging greater than 5° at 3 consecutive vertebral levels [4]. Although a distinct cause is unknown, it is postulated to arise from an injury to the vertebral growth plate during the adolescent period, causing cessation of further development [4]. Scheuermann's Disease can lead to thoracic hyperkyphosis, which may ultimately place increased strain at the thoracolumbar and cervicothoracic junctions. This is supported by evidence of increased disc pathology at transitional areas like the midthoracic (T7–T8) spine and thoracolumbar junction (T11-L1) [4].

Although a growing amount of literature has tested conservative treatments for idiopathic scoliosis [5-11], conservative treatments for scoliosis secondary to bony or soft tissue developmental disorders have not been widely tested in the chiropractic literature.

This paper discusses the results of 3 clinical patients with scoliosis and their respective case histories, treatment, and results. The first case describes the treatment of a patient with a past history of surgical stabilization. While there is some information available regarding chiropractic treatment of scoliosis, we could find no published reports detailing treatment of a scoliosis patient while surgical hardware was still in place. The second case involves a male with scoliosis secondary to Scheuermann's Disease. Finally, the third case details the history and treatment of a female with a rare left thoracic-right lumbar scoliosis pattern.

## Case presentation

### Case #1

#### History and examination

A 37-yr-old female presented to a private spine clinic with a chief complaint of episodic neck and back pain. The subject began care while her daughter was being treated for scoliosis in the same clinic. She presented with a past medical history including previous diagnosis and treatment for adolescent idiopathic scoliosis. Her previous treatment included spinal fusion and Harrington rod instrumentation. Preoperatively, a 58° right thoracic scoliosis was found between T6 and T11. Harrington rod instrumentation reduced the scoliosis to 26°. We were unable to review her medical records pre and post arthrodesis. Although her family history identified a possible genetic component with her daughter's medical history, her preceding family history was negative for scoliosis.

The subject initially filled out a Functional Rating Index. This index, described and tested by Feise et al [12], is a combination of the Neck Disability Index and the Oswestry Back Pain Index. This form provides a valid and reliable self-rated assessment of functional improvement in daily activities.

On static visual posture examination, a moderate anterior right shoulder, a protruding right scapula, and a right rib hump were identified. These visual postural findings are used as screening indicators so that unnecessary radiographic studies are not undertaken. Adam's test confirmed the right rib hump on forward bending. This test is classically used in the primary care setting to screen for scoliosis, although its reliability has been called into question [13].

**Figure 1:**
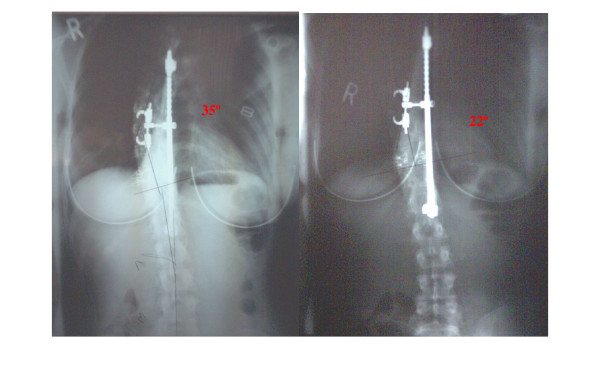
This figure shows the pre and post AP lumbodorsal radiographs. This patient, following 8 office visits in 8 weeks, obtained an apparent Cobb angle reduction of 13° when measured from superior of T6 to inferior of T11.

The radiographs series consisted of lateral cervical and lumbar views, as well as opposing frontal views. The lateral films were taken to calculate the amount of cervical lordosis, forward head posture, and lumbar lordosis. The cervical lordosis was measured from an angle between 2 lines intersecting the posterior C2 and C7 vertebral bodies. The lumbar lordosis was taken from the angle formed by the intersection of 2 posterior tangent lines drawn from the back of L1 and L5. Preliminary evidence suggests that correcting the sagittal spine before reducing the scoliotic curvature may promote a longer lasting correction [14,15]. In this case, the initial cervical lordosis measured 23° from C2 to C7, the initial forward head posture measured 31 mm, and the lumbar lordosis measured 31°. Analysis of forward head posture was performed by drawing a vertical line from the posterior inferior corner of C7 upward [16]. The distance from this line to the posterior superior corner of C2 is measured in millimeters. The initial standing AP radiograph showed a right thoracic scoliosis of 35°, shown in Figure 1. This measurement was taken from a Cobb angle drawn between the superior endplate of T6 and the inferior endplate of T11. We used a sectional view of the thoracolumbar spine to reduce positional distortion commonly encountered on full-spine films [17]. The film was taken at a 72" film to focal distance (FFD) to reduce magnification distortion. For radiographic analytical purposes, we used the positioning and analysis methods outlined by Harrison et al [16,18-21]. These methods have shown good to excellent reliability in terms of patient positioning, and inter- and intra-examiner reliability. Initially, the patient self-rated her back and neck pain as a 7/10 on a numerical pain rating scale.

#### Intervention and outcome

The Pettibon corrective procedures [22] were used in this patient's care plan. The goal of these procedures is to promote a normal [23-25] sagittal spinal contour. A specific treatment plan was created based upon a trial treatment involving the Pettibon procedures. The patient received bilateral cervical spine traction-type manipulation to mobilize several cervical spinal joints, and then was immediately fitted with a 4-lb Pettibon Headweight. ^® ^The patient walked on a treadmill for 10 minutes while wearing the headweight. After 10 minutes, a follow-up lateral cervical radiograph was taken while wearing the headweight. The purpose of this lateral stress view is to evaluate the potential improvement in cervical lordosis and reduction in forward head posture. Cervical lordosis and forward head posture are again measured on these stress views to evaluate response to treatment. Although earlier studies suggest that a 23° cervical lordosis may also be normal [26-28], newer research identifies a cervical lordosis closer to the 40° range [23,29,30] Despite this evidence; the concept of a normal cervical lordosis remains a debatable issue. Once it was determined that the patient could benefit by the proposed treatment, a plan was developed and implemented specifically for her. Her plan included once-weekly office visits, with an emphasis on home care exercises to promote patient independence. Each visit consisted of warm-up procedures, manipulation, and rehabilitative exercises.

**Figure 2:**
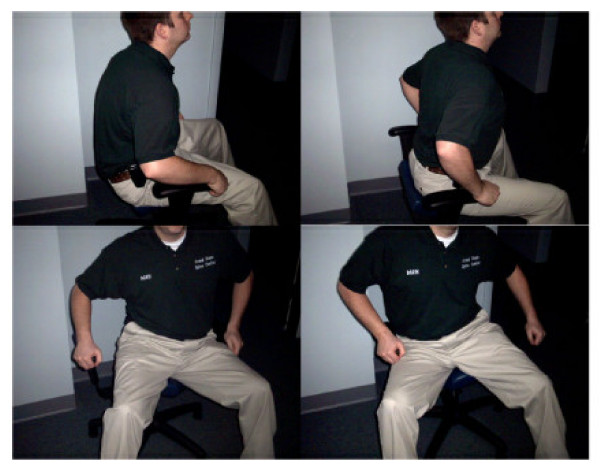
The figure demonstrates the warm-up procedures performed prior to each manipulative treatment. The patient performs a series of exercises, starting front-to-back, side-to-side, clockwise, and counterclockwise motions. All three patients performed these warm-ups at each office visit.

The warm-up procedures consisted of Pettibon Wobble Chair^® ^Exercises, shown in Figure 2. The Pettibon Wobble Chair^® ^is a chair designed to isolate the lumbar spine so that core training may take place. The goals of the chair are to promote lumbar stability, muscular coordination, and increase flexibility. However, the benefits of the chair itself remain to be investigated. The Wobble Chair^® ^exercises are performed by holding the head and shoulders still, moving only the pelvic girdle. The exercises consist of a front-to-back motion, a side-to-side motion, and clockwise/counterclockwise circles. Each exercise was performed 20 times, for a total of 80 repetitions at each office visit.

Side-posture lumbopelvic adjustments were delivered bilaterally to mobilize the sacroiliac joints. Cervical spine manipulation was performed by hand in accordance with the radiographic findings. The cervical spine manipulative procedures can be found in the osteopathic literature [31].

**Figure 3:**
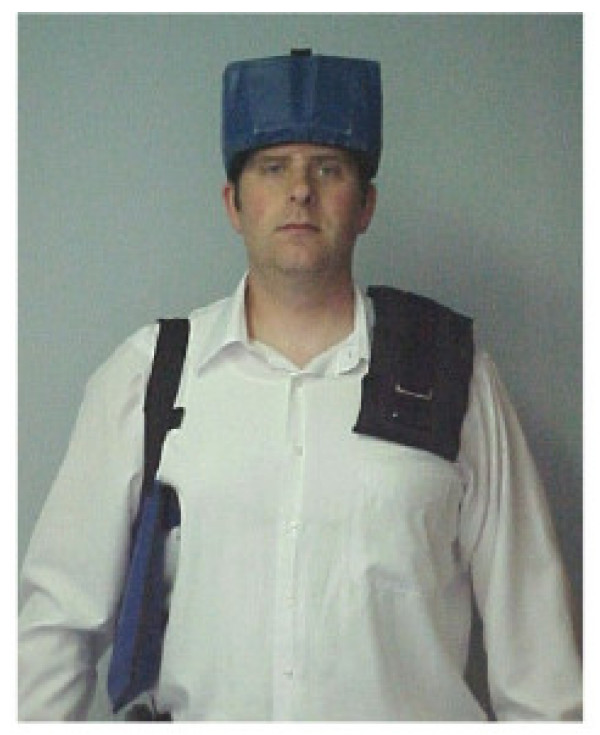
This figure provides a sample illustration of the placement for the proprietary weighting system. A headweight and shoulderweights are pictured.

The rehabilitative included the use of a 4-lb anterior Pettibon Headweight^®^, a right low shoulderweight, and a left high shoulderweight. An illustration of the weighting system is shown in Figure 3. During each office visit, the subject wore the headweight and shoulderweights while standing or walking. This exercise was performed for 10 minutes following the manipulative procedures. The patient was instructed to wear the headweight and shoulderweights at home for 20 minutes twice daily. Positional traction, on 2 triangular foam blocks placed at the cervicothoracic and thoracolumbar junctions, was performed once daily immediately before bed for 20 minutes.

After 8 visits in 8 weeks, post radiographs were taken to quantify changes in the sagittal and frontal spinal curves. Additionally, the subject filled out a follow-up Functional Rating Index to compare to the original. The Functional Rating Index score dropped from a 33% disability rating to 8%, and the numerical pain rating scale, rated a 7.0/10 at the onset of care, dropped to a 0/10. The average numerical pain rating scale score over the 8-week span was 3.3 out of 10.

On the post-treatment anteroposterior radiograph, the Cobb angle from T6–T11 was reduced from 35° to 22°. Her cervical lordosis measured 40°, while her forward head posture reduced to 13 mm. The follow-up radiographs were taken at the beginning of the 9^th ^visit prior to treatment, one week after the previous treatment.

### Case #2

#### History and examination

A 30-yr-old African-American male presented to a private spine clinic with a chief complaint of chronic mid thoracic pain. The patient had a previous medical diagnosis of Scheuermann's Disease. Moderate wedging was found on previous lateral lumbar and thoracic radiographs at the levels of T7–T10. The patient reported having the back pain consistently over the last 8–10 years, with recurrent episodes of intense myospasms occurring in the paraspinal musculature at the thoracolumbar junction. The patient had been previously managed unsuccessfully with prescription NSAIDS, muscle relaxants, and physical therapy consisting of cryotherapy, electric stimulation, and postural isotonic exercises. The patient could not recall any childhood traumatic events that may have contributed to the vertebral wedging asymmetry.

The subject initially filled out a Functional Rating Index [12]. We used this form to provide an objective assessment of functional improvement in daily activities. On static visual posture examination, a moderate high and anterior left shoulder and a right rib hump were identified. The paraspinal thoracolumbar musculature had also been significantly developed. Although these factors are not differential for Scheuermann's Disease, they do represent postural abnormalities often associated with scoliosis. Palpatory findings included marked areas of spasticity over the right latissimus dorsi, the left trapezius, the left quadratus lumborum, and the left rhomboid muscles.

Standing anteroposterior and lateral cervical and lumbar radiographs were obtained and analyzed for regional alignment as previously described. Gross radiographic visualization showed a postural swayback positioning, where the pelvis shifts anterior in relation to the thoracic cage. This may result from activation of the pelvo-ocular reflex to compensate for a forward head position [32]. The initial absolute rotation angles (ARA) from C2–C7 on the lateral cervical view [16] and L1–L5 on the lateral lumbar view [20] were drawn and measured. Prior to treatment, these angles measured 32° and 55°, respectively. According to Harrison et al, the normal lumbar lordosis should measure 39.7°, with a majority of the lordosis comprised in the L4-S1 region [25]. Prior to treatment, the forward head posture measured 22 mm, compared to an average normal of <20 mm [28]. The vertical axis line (VAL), measured from the anterior portion of the sacral base, should intersect the T11/T12 area [25]. In this case, the patient's VAL was 56 mm anterior to this interspace, consistent with a swayback type of posture. In the coronal views, a left thoracic scoliosis was found between the levels of T1–T5 measuring 22°. Nothing remarkable was found on the AP lumbopelvic.

The patient began an initial treatment plan consisting of 3 weekly visits for 4 weeks. The goals of this initial treatment plan were very specific, including restoring normal sagittal cervical and lumbar curves, reducing forward head posture, and reducing the swayback posture.

#### Intervention and outcome

The initial 4 weeks of care consisted of manipulative and rehabilitative therapy designed to improve the static alignment of the sagittal spine. These methods are part of the Pettibon system [33]. The first 12 visits entailed the same procedures in the same order. To begin each visit, the patient performed a series of exercises on a Pettibon Wobble Chair^®^. This chair is consists of a multiplanar seat that allows the user to perform specific spinopelvic motion patterns. Clinical observation by the authors suggests that these exercises seem to make the manipulative treatment easier on the patient.

In this case, manipulative treatment included bilateral cervical manipulation and anterior thoracic manipulation to mobilize any restricted cervical and/or thoracic segments.

Following the manipulative treatment, the patient was fitted with a Pettibon Headweight^® ^containing 4 lbs on the front of the forehead. The patient walked for 15 minutes while wearing the headweight. After 15 minutes, the patient laid supine on a pair of high-density foam blocks to promote a normal sagittal spinal contour. This was done while lying on an intersegmental traction table for 7 minutes. The patient was prescribed specific home care exercises to be performed daily between visits, and was instructed to walk with the Pettibon Headweight^® ^for 20 minutes twice daily on non-clinic days, and lie on the high-density foam blocks for 20 minutes every night immediately before bed. After 4 weeks, post treatment lateral cervical and lateral lumbar radiographs were taken to quantify improvement in sagittal alignment.

The post lateral cervical showed a 32° cervical lordosis and 5 mm of forward head posture. The post lateral lumbar showed a 44° lumbar lordosis, while the vertical axis line fell 30 mm from the T11/T12 interspace. The 4-week functional rating index improved from a 70% disability to 50% disability, while the numerical pain rating scale dropped from a 9/10 to an 8/10.

**Figure 4:**
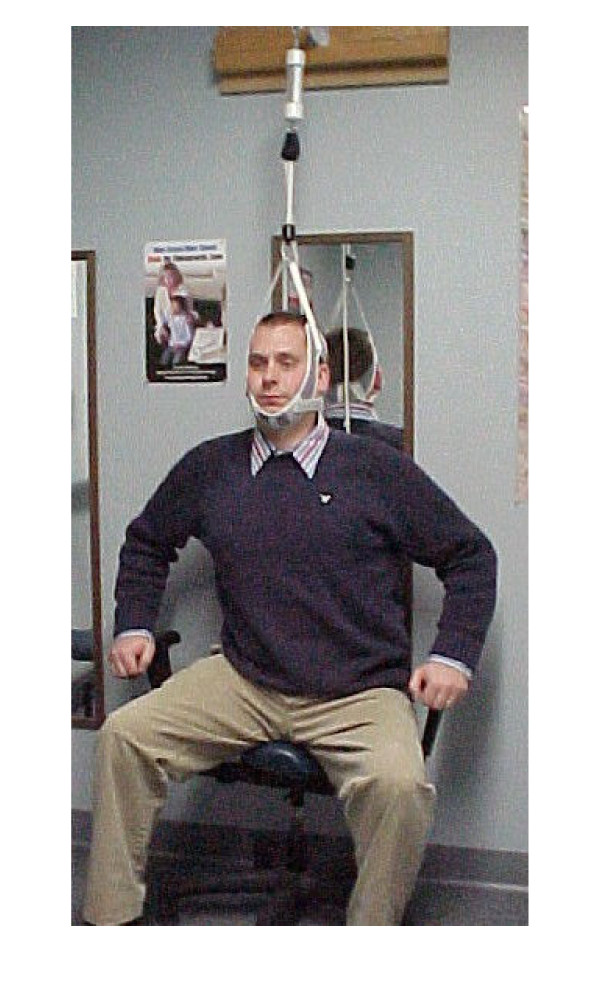
This figure illustrates the combined of cervical traction and the Wobble Chair exercises. This procedure was performed after each manipulative treatment.

**Figure 5:**
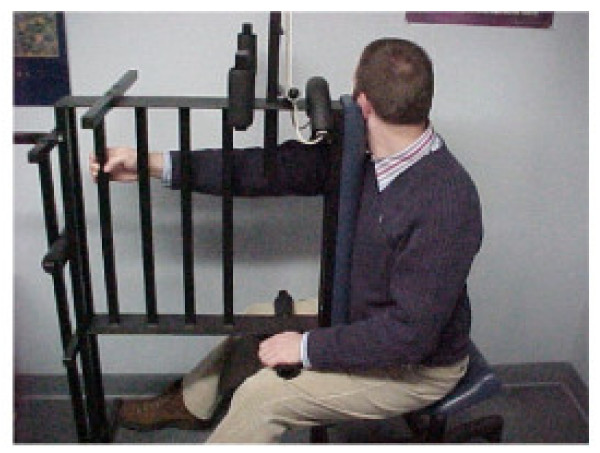
The rhomboid pull is demonstrated here in Figure 4. The goal of this exercise is to change the origin and insertion of the isolated rhomboid muscle. This is used in attempts to de-rotate the spine toward the rhomboid.

**Figure 6:**
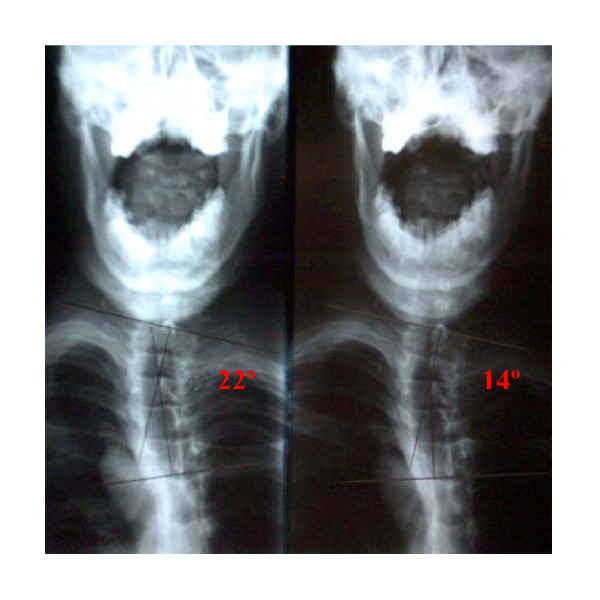
This figure displays comparative AP cervicothoracic views, taken initially and after 20 weeks of treatment. Despite the presence of bony deformity, a Cobb angle reduction from 22° to 14° was still obtained, although the frequency of care was higher than the other 2 cases.

Given the presence of bony deformity, we felt that significant time must be spent reducing the asymmetrical loading in the thoracic spine for coronal correction to be achieved. Therefore, the frequency of visits remained at 3 times per week over the next 20 weeks. Over this 20-week period of care, the manipulative treatment remained the same. However, several new rehab procedures were added. The patient still wore the headweight for 15 minutes immediately following the manipulative treatment. After the headweight, the patient worked out on the Pettibon Wobble Chair^® ^while simultaneously performing cephalad traction, demonstrated in Figure 4. Following this procedure, a specific isometric exercise was performed on a Pettibon Linked Trainer. ^® ^This exercise, shown in Figure 5, is designed to isolate the right rhomboid muscle. Theoretically, the linked trainer stabilizes the scapula, thereby functionally changing the origin and insertion of the rhomboid. This form of exercise has been previously illustrated with practitioner assistance [34]. Typically, the function of the rhomboid is to retract the scapula. However, when the scapula is stabilized, now the muscle may effectively pull on its proximal attachment, that being the spinous processes from T5–T8. Therefore, by switching the action of the muscle, our goal was to use the rhomboid to help reduce the left thoracic scoliosis. The patient was instructed to perform this exercise by pulling and holding for 10 seconds, repeating this process until the muscle is sufficiently fatigued. Finally, lateral traction was performed on the thoracic scoliosis using a high-density foam block while in a side-lying position. This block was placed beneath the apex of the scoliotic curvature for 15 minutes. Home care exercises remained the same. However, the frequency of the exercises was dropped to 3 times per week instead of daily. At the conclusion of the 20 weeks, post treatment AP cervicothoracic and lumbopelvic radiographs were taken to quantify improvement. The Cobb angle of the left thoracic scoliosis from T1–T5 reduced to 14°. A comparative view of the pre and post AP cervicothoracic views is shown in Figure 6. A 20-week functional rating index score dropped to a 28% disability rating, while the numerical pain rating scale dropped to a 6/10.

### Case #3

#### History and examination

A 23-year-old female presented with bilateral diffuse neck and lumbodorsal pain, and right-sided scapular and shoulder pain. The pain was constant and sharp in nature with radicular pain into the right arm and elbow. At age 12, her primary care physician diagnosed her with adolescent idiopathic scoliosis. At that time, the treatment plan was mainly comprised of observational methods, such as radiographs, visualization, and MRI. About one year before presenting to the primary author's clinic, she was referred for physical therapy by an orthopedic surgeon, which produced little subjective benefit, according to the patient.

On visual examination, a prominent left posterior rib hump was identified. In the frontal plane, she also displayed a marked high left shoulder with anterior rotation. Left anterior pelvic rotation was also well visualized. Given these preliminary findings, along with the positive past history of scoliosis, radiographic imaging was ordered to locate and calculate the nature and severity of the scoliosis. Initial standing 14" × 17" sectional radiographs showed a 37° left thoracic scoliosis, measured from the superior endplate of the T3 vertebra and the inferior endplate of the T7 vertebra. She also had a 26° right lumbar scoliosis measured from the superior endplate of T10 and the inferior endplate of L3. In the sagittal plane, her initial cervical lordosis measured 18°, while her lumbar lordosis measured 50°.

#### Intervention and outcome

The patient began a treatment plan of 3 visits per week for 4 weeks, followed by once weekly visits for 12 weeks. Goals for the first 4 weeks of treatment included: 1) improvement of sagittal spine alignment, 2) reduction in pain and symptoms, and 3) functional improvement. A specific treatment routine was followed at each visit for the first 12 visits.

Each visit began with spinal warm-up exercises performed on a Pettibon Wobble Chair™. The patient then received a brief (less than 15 minutes) session of deep tissue massage therapy applied to the postural muscles. Following these procedures, manipulative intervention took place. The manipulative techniques are collectively taught within the Pettibon technique [33], and were employed according to this methodology. First, a posteroanterior high-velocity, low amplitude (HVLA) procedure was applied to mobilize the thoracolumbar region. This was followed by anterior thoracic manipulation to mobilize the cervicothoracic region. A side-lying sacral manipulation was performed bilaterally to mobilize the sacroiliac joints and the lumbosacral joint. Cervical manipulation was performed only on those visits where a supine leg check revealed evidence of leg length inequality (LLI). In the cervical region, an HVLA thrust was applied cranially, thus creating a traction-type adjustive force compared to more traditional shear- or rotary-type cervical manipulative procedures. All of the manipulative techniques are well illustrated and explained by Gibbons and Tehan [31]. The patient received cervical manipulation in 8 of the first 12 visits.

Immediately following the manipulative intervention, the patient performed her spinal rehabilitative care. In her case, a 4-lb Pettibon Headweight was worn on the front of the head for 10 minutes while maintaining a standing position. Finally, the patient ended each of these visits with the supine positional traction for 7 minutes. The patient was instructed to perform the headweight twice daily between visits for 20-minute intervals. She was also given a set of foam blocks to lie on at night for 20 minutes immediately before bedtime.

After this initial 4-week treatment period, a follow-up radiographic series was obtained, along with a follow-up Functional Rating Index. Comparative radiographic analysis showed a reduced Cobb angle of 29° from T3–T7 and 18° from T10-L3. The sagittal cervical lordosis improved to 32°, while the lumbar lordosis decreased to 45°. The follow-up Functional Rating Index score dropped from 48% to 28% disability.

Following this treatment period, clinical visits dropped to once weekly over the next 12 weeks. During this time, the Pettibon Linked Trainer™ was incorporated into her treatment plan. The Linked Trainer™ exercises were performed after the anterior headweighting procedure at each visit. Dynamic cervical traction was also applied while performing the Pettibon Wobble Chair™ exercises, immediately prior to the spinal manipulative therapy. Finally, a side-lying traction procedure was added to her treatment to help lengthen the soft tissue structures on the concave side of the spinal curvatures. A triangular foam block was placed under the patient's left side, below the apex of the thoracic curvature, while a 25-lb weight was placed above the apex of the lumbar curvature. The patient assumed a left side-lying position during this traction session. This traction maneuver followed the anterior headweighting and the Linked Trainer™ exercises. This procedure was performed for 40 minutes at each office visit as well as at home once daily. The frequency of headweight use at home dropped to 3 days weekly instead of daily.

**Figure 7:**
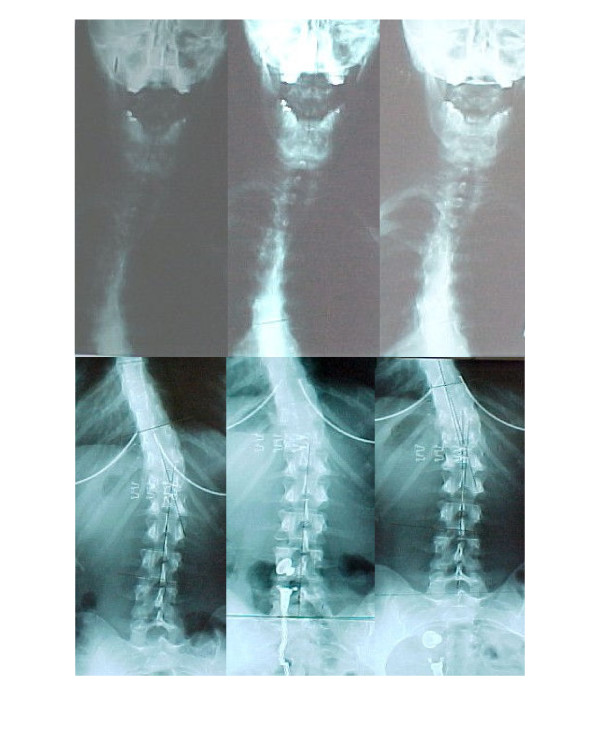
This figure shows the radiographic progress after the various stages of treatment.

After 12 weeks of the foregoing treatment, the patient was again re-evaluated using static spinal radiography and the Functional Rating Index. Radiographic analysis demonstrated a 21° left thoracic scoliosis from T3–T7, and a 15° right lumbar scoliosis from T10-L3. Her Functional Rating Index score further reduced to an 18% disability. The patient was asked to continue once daily home treatment consisting of the side-lying traction procedure for 40 minutes, and supine positional traction 20 minutes immediately before bedtime. She was also instructed to continue wearing the anterior headweight at home 3 days a week for 15 minutes per day. After 10 months under this home care regimen, the patient presented for a second follow-up evaluation. At this time, her Functional Rating Index reduced to an 8% disability, while her sagittal cervical and lumbar curves marginally improved to 34° and 42°, respectively. Her left thoracic scoliosis was further reduced to 18°, and her right lumbar scoliosis was maintained at 15°. Therefore, after a total of 4 months of active treatment and 10 months of weekly home care rehabilitation, her spinal curvatures were reduced a total of 19° in the thoracic curvature and 21° in the lumbar curvature. Her pre- and post- radiographs are shown in Figure 7.

## Discussion

Detailed reviews by Harrison et al [35-37] and Rhee et al [14] suggest that preserving a normal sagittal spinal contour may be important for long-term health. De Jonge et al [15] described how correction of lateral scoliotic curvatures caused a spontaneous restoration of the sagittal spinal curves, suggesting that loss of sagittal spinal curves may somehow be related to scoliotic curvatures.

Scoliosis places otherwise symmetrical muscle groups under longstanding, isometric, asymmetrical loads [38-41], which may compromise circulation within the muscle, ultimately leading to myofascial trigger points and chronic inflammation [42]. Weinstein et al [3] reported that scoliosis patients may retain high levels of function in later life, but do report higher instances of chronic back pain.

In addition to higher instances of chronic back pain, significant psychological issues may arise from concern over cosmesis and conventional treatment. Freidel et al [43] measured the self-perceived quality of life in women with scoliosis using the SF-36 questionnaire. They concluded that the psychosocial aspects of scoliosis and scoliosis treatment should be addressed in the treatment of this group of patients. Similarly, Sapountzi-Krepia et al [44] described the psychological distress that adolescents encounter while going through bracing treatment for scoliosis. A case-control study by Danielsson et al [45] identified a potential negative impact on the ability to function sexually due to conventional treatment restraint or self-consciousness of physical appearance.

Aside from back pain and psychological disturbance, several studies also suggest that scoliosis affects more than the musculoskeletal system. Curvatures of the thoracic spine are associated with restrictive lung disease due to ribcage deformity and decreased chest wall compliance [46]. Chest wall compliance is inversely proportional to the magnitude of the Cobb angle down to 10°, and vital capacity is reduced by decreased chest wall compliance directly [46,47]. Exercise endurance is also inversely diminished with increasing Cobb angle, even in patients with normal resting vital capacity [48]. Thoracic scoliosis may also cause shortness of breath and recurrent respiratory infections [46,49]. Indeed, scoliosis affects more than the musculoskeletal system.

Concerning coronal Cobb angle measurement for scoliosis, manual radiographic measurement has consistently shown good to excellent inter- and intra-observer reliability [50-53]. Previous studies demonstrate a manual Cobb angle measurement error on full-spine radiographs of 2.5 – 4.5° [51-53]. However, to achieve this low error, it is imperative that the same end vertebrae, same protractor, and same endplates are consistently chosen. Importantly, these measurement errors were extracted from data collected on full-spine radiographs. Patient positioning can significantly negatively impact measurements on full-spine radiographs [17]. The Cobb angle measurements in our study were taken from sectional radiographs, which reduce the positional distortion caused by inconsistent patient positioning. It is unknown to what extent the use of sectional radiography has on Cobb angle measurement error, if any.

The treatments outlined here required home care exercises, as described earlier. However, these exercises, which take up a combined 60 minutes per day, can be done in private, away from scrutiny by peers, neighbors, or relatives. This is in contrast to bracing treatment, where the brace must be worn at least 18 hours per day to achieve a good clinical result [54].

We placed the headweight, shoulderweight, and hipweights in areas designed to reduce our patient's specific spinal distortion patterns on radiograph. The patient was evaluated radiographically while wearing the headweight and shoulderweights to determine optimal position and weight. Our repeated clinical observation has demonstrated that patients may visually appear to improve with a shoulderweight in a certain position. However, they can look dramatically different on radiograph (migration away from the vertical axis) than they appear in visual posture analysis. This is consistent with recent failed attempts to objectify visual posture analysis as a valid clinical tool [55]. It is prudent to develop alternative methods of evaluation to avoid unnecessary radiation exposure to patients.

Because of the anterior wedging from T7–T10 in case #2, it is not surprising that over time a thoracic hyperkyphosis and swayback developed in this patient. As a result, marked anterior weight bearing of the head was required to maintain a horizontal eye level, thus satisfying the postural reflexes [56-61] Additionally, the marked forward head posture elicits the pelvo-ocular reflex, causing a forward shift of the pelvic girdle under the forward head position [32]. Therefore, the postural distortions seen in this case may represent compensatory changes over time as a result of thoracic buckling, a posture known to commonly cause increased mechanical stress at the spinal transition areas [4,24]. Correcting these compensatory postural changes proved to be a challenge, given that the impetus for them (the anteriorly wedged thoracic vertebra) could not be immediately, if ever, changed. However, within the confines of the Hueter-Volkmann law, we postulate that sustained correction of the asymmetrical mechanical spinal loading may theoretically help these vertebrae to remodel to some degree. Although the forward head posture is a compensatory reaction to the hyperkyphosis, the cervical spine soft tissue has likely remodeled to the forward head posture, given the likely duration of its existence [62]. Therefore, we felt that direct correction of the forward head posture must also be achieved to improve overall sagittal alignment, given the neurological control and importance of head position on upright spinal position [63]. This hypothesis remains to be definitively evaluated.

The significance of cases #2 and #3 lies in the location of the scoliotic curvatures. In the vast majority of cases, double major curvatures usually maintain a right thoracic/left lumbar pattern. In this case, the pattern was reversed, showing a left thoracic/right lumbar scoliosis. Several authors have previously discussed the unique presence of a left thoracic – right lumbar curvature pattern. McCarver et al [64] showed that only 1% of 550 patients with idiopathic scoliosis had double major curvatures consisting of a left thoracic – right lumbar configuration. Winter and Lonstein [65] maintained that any left thoracic curvature should be further evaluated for neurological abnormalities, such as neurofibromatosis, spina bifida, or syringomyelia. Finally, Schwend et al [66] also concluded that additional testing was necessary in left thoracic curvatures, given an observed higher incidence of neurological clinical signs. Case #3 seems to correlate these findings given the left thoracic scoliosis secondary to Scheuermann's Disease. It is important to note, however, that treating the Scheuermann's Disease itself was not our aim. Rather, our goal was to reduce the thoracic scoliosis secondary to it. We are not attempting to show that this treatment may affect the Scheuermann's Disease. In this case, however, additional testing was conducted at the initial time of discovery of the scoliosis. Further, my initial neurological examination also failed to produce any remarkable neurological findings.

Recently, several authors have discussed the relationship between the sagittal spinal contour and scoliosis [14,15,67,68]. Harrison et al [35-37] have discussed the pathophysiologic changes associated with the loss of the sagittal curves. Based on this evidence, we decided that it was important to the long-term outcome to address these spinal parameters.

Cases #1 and #2 present what appears to be inconsistent findings. Case #1 initially had a 23° cervical lordosis, below asymptomatic 31–40° range identified by McAviney et al [30], and the normal 34° identified by Harrison et al [28]. However, case #2 displayed a 32° initial cervical lordosis despite having a thoracic hyperkyphosis. In case #1, the patient had 31 mm of forward head posture. Since forward head posture reduces the magnitude of the cervical lordosis [69,70], a 23° cervical lordosis may not be normal for this patient. Additionally, recent evidence suggests that sagittal balance may more closely correlate to symptoms than sagittal alignment [71] Cervical lordosis by itself may not provide an accurate assessment of normal for each patient. Therefore, we suggest that both the cervical lordosis and forward head posture be weighed before a patient's cervical spine may be considered "normal." In contrast, case #2 had a both a normal cervical lordosis and forward head posture (32° and 22 mm, respectively). Therefore, we classified this patient's cervical spine as normal, despite the thoracic hyperkyphosis. We feel that the 55° lumbar hyperlordosis is a direct compensation for the swayback posture created by the thoracolumbar vertebral remodeling. This is consistent with the post treatment reductions in the swayback posture and lumbar lordosis.

In the Pettibon system, most of the manipulative treatment is not administered on a vertebral segmental basis. Rather, it is delivered to a specific region of segments so that the entire region may be mobilized. The goal of manipulative therapy in the Pettibon system is to mobilize several vertebral joints so that the rehab procedures can target the joints while they temporarily have an increased range of motion [33].

The purpose of the Pettibon Weighting System™ is to artificially alter the centers of mass of the head, trunk, and pelvis, causing reactive corrections by the postural reflexes [72-74]. The goal of postural reflexes is to maintain efficient body stance and locomotion using the least energy expenditure possible [56,63,75]. In the present cases, each patient was instructed to continue with their home exercise routine on a once weekly basis in attempts to maintain the change in spinal configuration.

The procedures that comprise the Pettibon system have been previously examined in specific clinical cases [5,76]. Although these techniques have been investigated for preliminary treatment of idiopathic scoliosis [5], they have not, until this point, been used in cases of scoliosis due to structural deformity or left thoracic primary curvatures. Given the perceived results of the cases outlined here, it is worthy of future investigations in such cases. However, case reports and case series designs do not provide substantive evidence of therapeutic effectiveness. This remains the realm of properly conducted prospective clinical trials.

Conservative treatment for scoliosis needs to be examined much more closely in the biomedical literature, as side effects [44-46] and compliance issues [54] make conventional treatments such as bracing less attractive to patients and parents of minor patients.

## Conclusion

In this case series, we reported the clinical results for 3 distinct types of scoliosis patients. While no firm conclusions relative to cause and effect can be made from these results, the moderation of the spinal curves may have merit. Although reductions in self-rated disability and pain scores were reported, they may not be attributable to the improvement in spinal alignment. Further investigation is required to determine the potential benefits of sagittal spine alignment in the correction of scoliosis and other health benefits.

## Competing interests

MM is the Director of Research for the Pettibon Institute, Inc. However, this is a volunteer position and he is not financially compensated by the institute in any fashion. The Pettibon Institute covers the research costs for MM, including literature reviews, statistical services, etc. TJ has no competing interests.
